# Functional activity of *E. coli* RNase R in the Antarctic *Pseudomonas syringae* Lz4W

**DOI:** 10.1186/s43141-023-00553-2

**Published:** 2023-10-16

**Authors:** Ashaq Hussain, Malay Kumar Ray

**Affiliations:** Centre for Cellular and Molecular Biology, Hyderabad, India

**Keywords:** Psychrophiles, Exoribonuclease R, Cold-adapted enzymes, Degradosome, Functional complementation, RNA processing, Catalytic domain

## Abstract

**Background:**

In Antarctic *P. syringae* RNase R play an essential role in the processing of 16S and 5S rRNA, thereby playing an important role in cold-adapted growth of the bacterium. This study is focused on deciphering the in vivo functional activity of mesophilic exoribonuclease R and its catalytic domain (RNB) in an evolutionary distant psychrophilic bacterium *Pseudomonas syringae* Lz4W.

**Results:**

Our results confirm that *E. coli* RNase R complemented the physiological functions of the psychrophilic bacterium *P. syringae* RNase R and rescued the cold-sensitive phenotype of *Pseudomonas syringae* ∆*rnr* mutant. More importantly, the catalytic domain (RNB) of the *E. coli* RNase R is also capable of alleviating the cold-sensitive growth defects of ∆*rnr* mutant as seen with the catalytic domain (RNB) of the *P. syringae* enzyme. The Catalytic domain of *E. coli* RNase R was less efficient than the Catalytic domain of *P. syringae* RNase R in rescuing the cold-sensitive growth of ∆*rnr* mutant at 4°C, as the ∆*rnr* expressing the RNB^Ec^ (catalytic domain of *E. coli* RNase R) displayed longer lag phase than the RNB^Ps^ (Catalytic domain of *P. syringae* RNase R) complemented ∆*rnr* mutant at 4°C. Altogether it appears that the *E. coli* RNase R and *P. syringae* RNase R are functionally exchangeable for the growth requirements of *P. syringae* at low temperature (4°C). Our results also confirm that in *P. syringae* the requirement of RNase R for supporting the growth at 4°C is independent of the degradosomal complex.

**Conclusion:**

*E. coli* RNase R (RNase R^Ec^) rescues the cold-sensitive phenotype of the *P. syringae* Δ*rnr* mutant. Similarly, the catalytic domain of *E. coli* RNase R (RNB^Ec^) is also capable of supporting the growth of Δ*rnr* mutant at low temperatures. These findings have a vast scope in the design and development of low-temperature-based expression systems.

**Supplementary Information:**

The online version contains supplementary material available at 10.1186/s43141-023-00553-2.

## Background

**Fig. 1:**
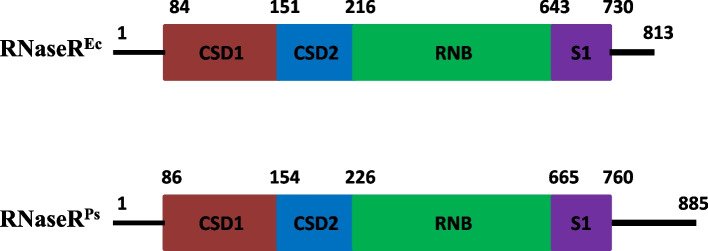
Domain organization of RNase R. Schematic representing the domain organization in *E.coli* and *P. syringae* RNase R. Individual domains are shaded in separate colors and numbers represent the amino acids in the primary sequence of protein

RNase R is a conserved hydrolytic ribonuclease (3' to 5') that belongs to RNase II family of exoribonucleases. This processive enzyme is capable of degrading the RNA molecules through their complex secondary structures, unlike the exoribonuclease RNase II, which can degrade only single-stranded RNAs [1–4]. The domain arrangement of RNase R is similar to that of RNase II and Rrp44, with the CSD1, CSD2, and S1 domains capping the top of the RNB domain [1, 5] (Fig. 1). The crystal structure of *E. coli* RNase R revealed a unique feature that differs from the crystal structures of other reported exoribonucleases is the presence of two open channels that act as important RNA binding sites, i.e., a top channel between the S1 and CSD1 domains and a side channel between the RNB and CSD1 domains [1, 6–8]. Mutational analysis of the enzyme has established that the aspartic acid residues D272, D278, and D280 in the catalytic pocket of the *E. coli* RNase R (RNase R^Ec^) are important for the ribonuclease activity, and D280 is directly involved in the catalysis during the hydrolytic cleavage of terminal nucleotides from the RNA chain. Further, the residue Y324 plays a key role in deciding the final length of end-products. The substrate binding domains (CSD1, CSD2, and S1) bind only to single-stranded 3′-overhang ends of RNA molecules, thereby screening the substrates and regulating the entry of RNA into the catalytic pocket of the enzyme [9]. Structural comparison of *E. coli* RNase R with other RNase II family proteins revealed two open RNA-binding channels in RNase R and suggested a tri-helix ‘wedge’ region in the RNB domain that may induce RNA unwinding. Construction of two tri-helix wedge mutants revealed that mutants, indeed lost their RNA unwinding but not RNA binding or degrading activities. Structural studies revealed that duplex RNA with an overhang is bound in the two RNA-binding channels in RNase R. The 3′ overhang is channeled into the active site and duplex RNA is unwound upon reaching the wedge region during RNA degradation [1].

Thus, the RNA binding domains CSD1, CSD2, and S1 play multiple roles in substrate recognition and sensing of 3′ overhangs of RNA molecules, whereas RNB domain plays a critical role in unwinding and processing/degradation of double-stranded RNA molecules [1, 2, 10].

Physiologically, RNase R plays a key role in the RNA metabolism of cell via degradation of polyadenylated RNAs, degradation of mRNA transcripts containing REP (repetitive extragenic palindromes) sequences [3], degradation of defective and non-functional tRNAs, trans-translation and quality control of ribosomes [11], turnover of ribosomal RNAs (e.g., 16S and 23S rRNAs) [8, 12–15] and growth-phase specific (e.g., stationary phase) removal of *ompA* mRNA in *E. coli* [16]. Depletion of RNase R affects tmRNA (transfer-messenger RNA) metabolism as evidenced by the accumulation of tmRNA precursors and tmRNA degradation intermediates [17]. The tmRNA-dependent trans-translation pathway is important for releasing the stalled ribosomes from truncated or defective mRNAs, tagging the proteins and peptides produced from the truncated mRNAs and facilitating the degradation of both defective RNAs and proteins. Thus, RNase R also plays a role in the maintenance of protein quality in the cells [18].

RNase R is a stress-induced protein that shows increased expression at low temperatures and stationary phase [16, 18]. In *E. coli* RNase R is encoded by *rnr* gene, and its levels in the cell are regulated by RNase G, RNase E, and SmpB [19, 20]. In *Streptococcus pneumoniae*, *Aeromonas hydrophila*, pathogenic *E. coli*, and *Shigella flexneri*, disruption of *rnr* gene leads to a decrease in virulence [21–23]. RNase R is also required for the low-temperature growth of *Legionella pneumophila* and *A. hydrophila* [24]. In *Mycoplasma genitalium* bearing the smallest bacterial genome, RNase R is the only exoribonuclease that plays a crucial role in all RNA metabolic processes including the processing and degradation of different types of RNA molecules [25–27]. Recent studies have illustrated that methylated ribose in the ribonucleotides acts as stop signals for RNase R-mediated RNA degradation [26]. This indicates that ribose methylation status of RNA might be used as a signaling mechanism by bacteria for the screening of RNA molecules to be sent for degradation or processing and maturation.

In our laboratory, RNase R was found to be a component of the novel RNA degradosomal complex of *P. syringae* Lz4W, in which RNase R  is associated with the endoribonuclease RNase E and RNA helicase RhlE [28, 29]. The disruption of *rnr* gene led to a cold-sensitive phenotype of the Antarctic bacterium. Further biochemical investigations revealed that cold-sensitive ∆*rnr* is defective in the 3′-end processing of 16S and 5S rRNAs, as a result of which *rnr* mutants accumulated unprocessed 16S rRNA and 5S rRNA precursor molecules in the cells [30]. Collectively, these studies have established three important things: first, psychrophilic bacteria like *P. syringae* Lz4W possess novel degradosome that has replaced the ss-RNA degrading exoribonuclease PNPase with the ds-RNA degrading enzyme RNase R in the protein complex; second, RNase R plays a crucial role in cold adaptation, and third, remarkably, the exoribonuclease enzyme plays a role in the 3′-end processing of 16S rRNAs which was hitherto unknown, but predicted to be an endoribonuclease mediated process [30].

RNase R is essential for growth of *P. syringae* at low temperature. *P. syringae* Δ*rnr* mutants display cold sensitive phenotype when grown at 4°C, whereas *E. coli* Δ*rnr* mutants do not display any low temperature associated growth defect. At molecular level, *∆rnr* mutants of *P. syringae* and *E. coli* accumulate rRNA degradation intermediates suggesting their role in rRNA degradation or quality control [28, 31]. However, *∆rnr* mutant of *P. syringae* accumulates 3′-end unprocessed 16S and 5S rRNA precursors at low temperature, while *E. coli* mutant does not. The *P. syringae* Δ*rnr* mutant is also defective in tmRNA degradation and processing. Thus, there were distinct similarities and differences in the activities and requirements of exoribonucleases (RNase R^Ps^ and RNase R^Ec^) during growth of the psychrophilic and mesophilic bacteria. Therefore, it was important to know whether *E. coli* RNase R would be able to complement the cold-sensitive growth defect of Δ*rnr* mutant of the Antarctic *P. syringae*.

The objectives of this study are (i) Will *E. coli* RNase R (RNase R^Ec^) complement the cold-sensitive phenotype of *P. syringae* Δ*rnr* mutant? (ii) Will the catalytic domain (RNB^Ec^) of *E. coli* RNase R be able to carry out similar in vivo functions like the catalytic domain (RNB^Ps^) of *P. syringae* RNase R despite their differences in in vitro activities?

Our study clearly demonstrates that the *E. coli*-specific exoribonuclease R (RNase R^Ec^) has retained all the functions that are necessary to support the growth of psychrophilic *P. syringae* at 4 °C. This is remarkable as *E. coli* itself does not grow at 4°C, and the enzymes in spite of their evolutionary divergence and alteration in the biochemical properties, have maintained the common essential activities. The individual catalytic domains (RNB^Ps^ and RNB^Ec^) are also functionally similar, as both of them are capable of supporting the growth of Δ*rnr* mutant at 4°C.

## Methods

### Growth media and bacterial cultures

The Antarctic *P.* *syringae* Lz4W was routinely grown at 22°C or 4°C (for optimum and low temperatures respectively) in Antarctic bacterial medium (ABM) composed of 5 g/l peptone and 2.0 g/l yeast extract, as described earlier [32, 33]. *E. coli* strains were cultured at 37°C in Luria–Bertani medium, which contained 10 g/l tryptone, 5 g/l yeast extract, and 10 g/l NaCl [34]. For solid media, 15 g/l bacto-agar (Hi Media) was added to ABM or LB. Both ABM and LB media were supplemented with ampicillin (100 μg/ml), kanamycin (50 μg/ml), and tetracycline (10 μg/ml) as per requirement.

Fresh ABM broth was inoculated with 1% of primary culture and incubated at 22°C or 4°C with constant shaking. The optical density of bacterial culture was measured after different time intervals at 600 nm [OD_600_] and plotted against time.

### Molecular biology methods

General molecular biology techniques including isolation of genomic DNA, polymerase chain reactions (PCR), restriction enzyme digestion and ligation, transformation, etc. were performed as described [32]. All restriction enzymes, T4 DNA ligase, and other enzymes used in this study were purchased from New England Biolabs (NEB). Plasmids were prepared by using a plasmid isolation kit (Qiagen). Oligonucleotides were purchased from a commercial source (Bioserve Biotechnology, India). Gene amplifications were carried out using high-fidelity pfx DNA polymerase (Invitrogen). The conjugal transfer of recombinant plasmids into *P.* *syringae* was carried out by a biparental mating method using the donor *E. coli* strain S17-1, as described earlier [35].

### Construction of plasmids for expression and complementation studies

All gene cloning experiments were performed in *E. coli* DH5α cells. The detailed methodology has been reported earlier [34, 36, 37]. All plasmids used for protein expression and genetic complementation are listed in Table 1.

**Table 1:** Plasmids used in this study

Plasmid	Description	Reference/source
pET28a	*Kan*^*r*^, Expression vector for N-terminal His-tagged proteins,	Novagen
pGL10	Broad-host cloning vector, IncP replicon, *mob*^+^*, Kan*^*r*^	[38]
pET28a*rnr*^Ps^	pET28a plasmid for over-expression of N-terminal His tagged *P. syringae* RNase R	[30]
pET28a*rnb*^Ps^	pET28a plasmid for over-expression of N-terminal His tagged RNB domain of *P. syringae* RNase R	[39]
pET28a*rnr*^Ec^	pET28a plasmid for over-expression of N-terminal His tagged *E. coli* RNase R	This study
pET28a*rnb*^Ec^	pET28a plasmid for over-expression of N-terminal His tagged RNB domain of *E. coli* RNase R	This study
pGL10*rnr*^Ps^	pGL10 expressing His-tagged *P. syringae RNase* R protein	[28]
pGL10*rnb*^Ps^	pGL10 expressing His tagged RNB domain of *P. syringae* RNase R protein	[39]
pGL10*rnr*^Ec^	pGL10 expressing His tagged *E. coli* RNase R protein	This study
pGL10*rnb*^Ec^	pGL10 expressing His-tagged only RNB domain of *E. coli* RNase R protein	This study

### Cloning and expression of *E. coli* RNase R

**Fig. 2:**
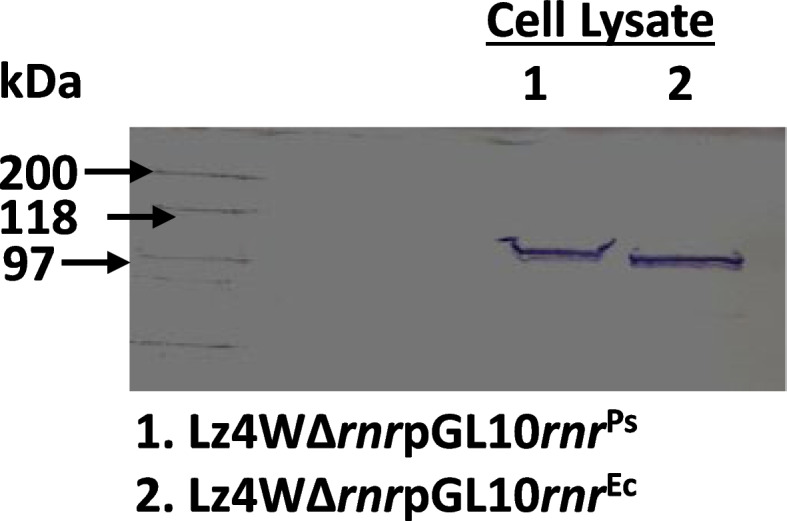
Expression of *E.coli* RNase R. Expression of RNase RPs and RNase REc in ∆*rnr* strain was analyzed by western blotting where cell lysate from ∆*rnr* strains expressing RNase RPs and RNase REc were transferred to a nylon membrane and probed by anti-His antibodies

The amplification and cloning of the RNase R encoding gene (*rnr*) of *P. syringae* has been reported earlier [30]. The *E. coli rnr* gene was amplified by using a set of gene-specific Forward and Reverse primers with incorporated *Nde*I and *Sal*I sites, and cloned into pET28a expression vector (Table S 1). The plasmid (pET28a*rnr*^Ec^) was transformed into *E. coli* BL-21 (DE3) strain, and expression of RNase R^Ec^ was observed under IPTG induction at various time intervals (Data not shown) [40]. The His-tagged *E. coli rnr* gene along with vector-specific RBS (Ribosome binding site) was released from pET28a*rnr*^Ec^ plasmid by *Xba*I and *Sac*I digestion and subcloned into broad host range vector pGL10 [38]. The construct (pGL*rnr*^Ec^) was transformed into *E. coli* S-17 strain and mobilized into *P. syringae ∆rnr* mutant by biparental mating as described earlier [35, 41]. Expression of the RNase R^Ec^ in *∆rnr* strain was confirmed by Western analysis using anti-His antibodies (Fig. 2).

### Cloning and expression of RNB domain of RNase R^Ec^

**Fig. 3:**
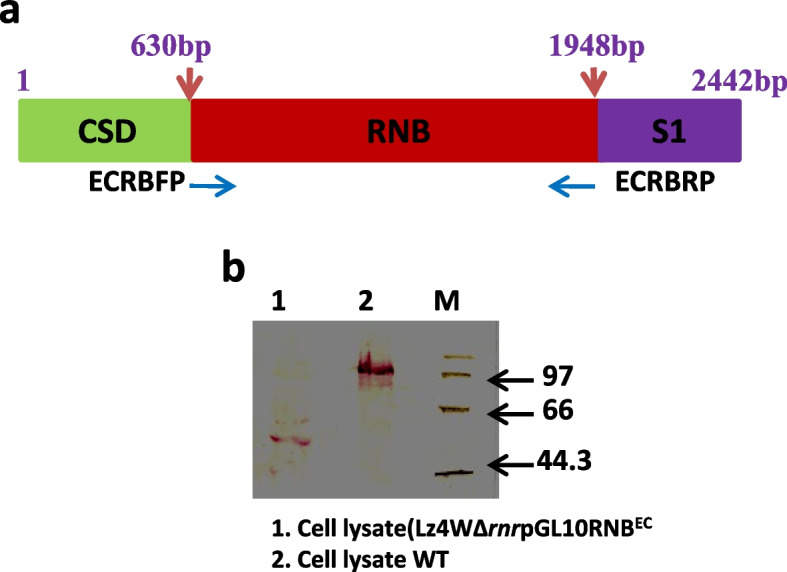
Expression of RNB (Catalytic) domain of *E. coli* RNase R.  (**a**) Color-coded schematic diagram showing different domains (color shaded) and position of primers employed for amplification of catalytic RNB domain. The numbers represent the nucleotide base pairs in *rnr* gene. (**b**) Expression of RNB domain in ∆*rnr* strain complemented by pGLRNBEc was analyzed by western blotting. Cell lysate from wild-type cells (Lane 2), ∆*rnr*pGLRNBEc strain (Lane 1) were loaded on polyacrylamide gel, transferred to a nylon membrane, and probed with anti RNase RPs antibodies

The truncated gene fragment (1239 base pairs) encoding catalytic (RNB) domain (413 amino acids) of *E. coli* RNase R was amplified using gene-specific forward and reverse primers with incorporated *Nde*I and *Bam*HI sites respectively, and cloned into pET28a expression vector. (Table S 1). The resultant plasmid pET28aRNB^Ec^ was transformed into *E. coli* BL-21(DE3) strain and expression of RNB^Ec^ domain under IPTG induction was confirmed by SDS-PAGE analysis (Data not shown). The fragment encoding the His-tagged RNB^Ec^ domain was released from pET28aRNB^Ec^ along with vector-specific RBS (Ribosome binding site) by *Xba*I and *Sac*I digestion and subcloned into broad host range plasmid pGL10 [38]. The construct (pGLRNB^Ec^) was transformed into *E. coli* S-17 strain and mobilized into *P. syringae ∆rnr* mutant by bi-parental mating as reported earlier [35, 41]. Expression of the RNB^Ec^ in *∆rnr* mutant was confirmed by Western analysis using anti-RNase R antibodies (Fig. 3).

### Functional complementation studies

Broad host range vectors pGL10*rnr*^Ec^ and pGL10*rnb*^Ec^ were mobilized into cold-sensitive ∆*rnr* strain, and growth pattern of complemented strains was analyzed at both optimal and low temperatures (22 °C and 4 °C). All bacterial strains used for genetic complementation studies are listed in Table 2.

**Table 2:** Bacterial strains used in this study

Bacterial strains	Description	Reference or source
*E. coli* DH5α	F- φ80*lac*ZΔM15 Δ(*lac*ZYA-*arg*F) U169 *rec*A1 *end*A1 *hsd*R17 (r_k_-, m_k_ +) *pho*A *sup*E44 λ- *thi*-1 *gyr*A96 *rel*A1 used for all gene cloning purpose	[36]
*E. coli* S17-1	*F _ pro recA1 (r_ m_) RP4-2 integrated (Tc::Mu) (Km::Tn7) [Smr Tpr]*; used as a donor strain in conjugation	[38]
*E. coli* BL21 (DE3)	*F*^*–*^*ompT gal dcm lon hsdS*_*B*_*(r*_*B*_^*−*^* m*_*B*_^*−*^*) λ(DE3 [lacI lacUV5-T7 gene 1 ind1 sam7 nin5])*, used for overexpression of proteins under IPTG induction	[40]
*P. syringae* (Lz4W)	Lz4W Amp^r^, wild-type	[29]
*∆rnr*	*rn*:: *tet*^*r*^ *P. syringae* strain with disrupted *rnr* gene	[30]
*∆rnr*(pGL10*rnr*^Ps^)	*∆rnr* strain complemented by *P. syringae rnr*	[30]
*∆rnr*(pGL10*rnb*^Ps^)	*∆rnr* strain complemented by only RNB domain of *P. syringae* RNase R	[39]
*∆rnr*(pGL10*rnr*^Ec^)	*∆rnr* strain complemented by *E. coli rnr*	This study
*∆rnr*(pGL10*rnb*^Ec^)	*∆rnr* strain complemented by only RNB domain of *E. coli* RNase R	This study

## Results

### Bioinformatic analysis of *P. syringae rnr* gene

Analysis of *rnr* gene (Gene encoding RNase R) sequences from different bacteria revealed that *rnr* locus is highly conserved among the *Pseudomonas.* sp. The operon consists of *rnr* (RNase R encoding) and *trmH* gene (encoding putative tmRNA or rRNA methyl transferase). Up-stream of the Bi-cistronic operon are two genes that code for tRNA-leucine, whereas downstream of the *rnr* operon is a highly conserved gene (*rpsF*) that codes for S6 ribosomal protein [39].

**Fig. 4:**
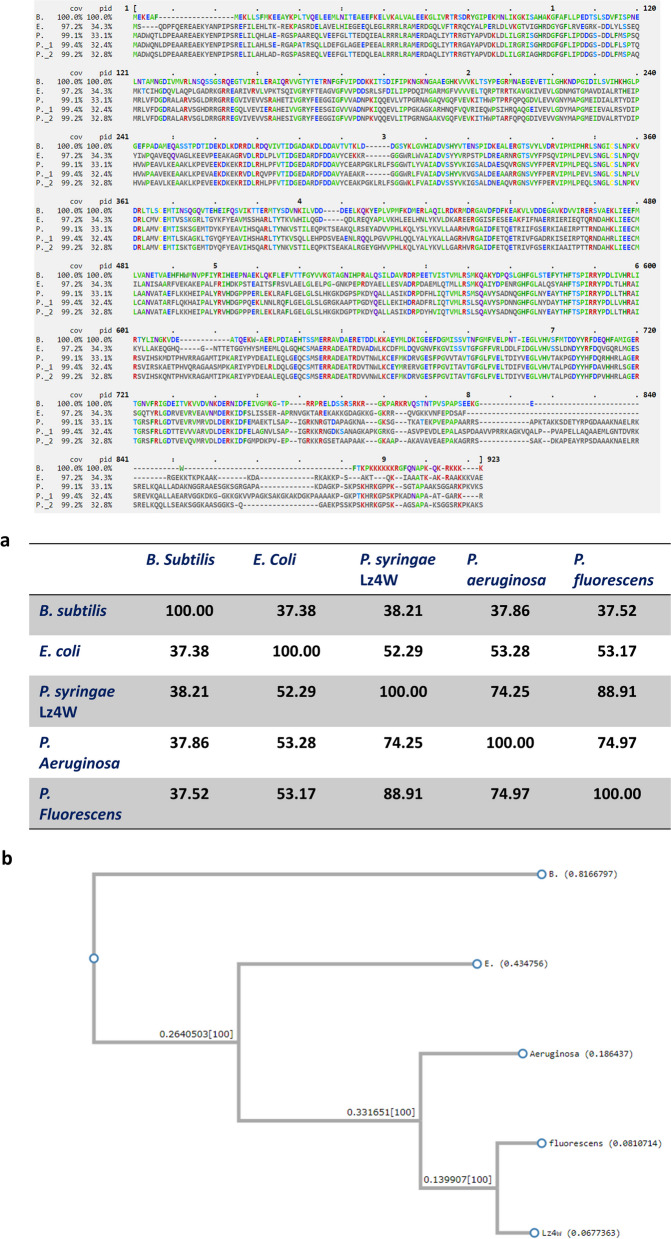
Multiple sequence alignment [T-coffee, www.ebi.ac.uk] of the amino acid sequence of the RNA helicases. Accordingly, *B. subtilis* has been indicated as B whereas *E. coli* has been abbreviated as E. Similarly *P. syringae* Lz4W, *P. aeruginosa*, and *P. florescens* have been indicated as Lz4W, P_1, and P_2 respectively. The alignment results also illustrate the identity among amino acid residues in different [N and C] regions of the protein

Sequence alignment studies (T-COFFEE, EMBL-EBI) of *rnr* gene from Antarctic *P. syringae*, *P. aeruginosa*, *P. fluorescens,* and mesophilic representative strains (*E. coli* and *B. subtilis*) revealed that *P. syringae* Lz4W displayed maximum similarity with *P. fluorescens* (88.91%), followed by *P. aeruginosa* (74.97%) (Fig. 4a). The Antarctic bacterium shows only a modest sequence similarity (53.17%) with *E. coli* and even lesser similarity with *B. subtilis* (37.52%)*.* Sequence alignment results illustrate that there is high similarity among the representative strains from *Pseudomonas* sp., as compared to their sequence similarity with representative strains from mesophiles (*E. coli* and *B. subtilis*).

A gene sequence-based phylogeny of exoribonuclease (*rnr*) genes from *Pseudomonas*. sp., and mesophilic *E. coli*, *B. subtilis* is shown in Fig. 4b. The analysis indicates that the *rnr* genes belonging to different strains have been clustered into three distinct groups representing, *Pseudomonas*. sp. (*P. syringae* Lz4W, *P. aeruginosa*, and *P. fluorescens*), *E. coli*, and *B. subtilis*. The representative strains from *Pseudomonas* group display sufficient homology among each other to be clustered as a group and provide a possible explanation for a convergent evolution among the representative strains in *pseudomonas* group or divergence from the mesophilic representatives which have been clustered in two separate groups.

### Growth analysis of ∆*rnr *strain complemented with RNase R^Ec^

**Fig. 5:**
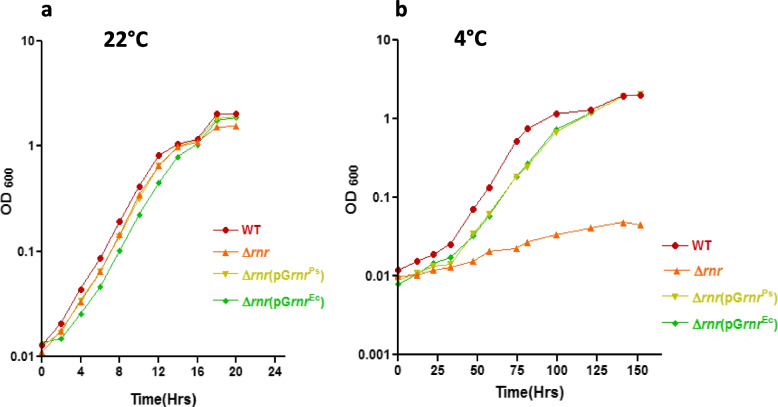
Mesophilic RNase R complements cold-sensitive phenotype of ∆*rnr.*** (a)** Growth profile of wild-type, ∆*rnr*, ∆*rnr*pGL*rnr*Ps, and ∆*rnr*pGL*rnr*Ec strains at 22°C and (**b)** at 4°C confirmed over-expression of RNase R^Ec^  from broad host range plasmid (pGL10) complements cold-sensitive phenotype of *Pseudomonas syringae* ∆*rnr* strain. For measurement of growth, samples were collected from each culture at regular intervals, OD at 600 nm was recorded and plotted against time. Each growth curve was performed at least three times

To confirm whether the *E. coli* RNase R^Ec^ is capable of complementing the cold-sensitive growth defect of *P. syringae ∆rnr* mutant, the complemented *∆rnr* mutant (*∆rnr*/pGL*rnr*^Ec^) expressing the *E. coli* RNase R was monitored for growth, and the growth profiles were compared with the wild-type and *∆rnr* mutant. Experimentally, all bacterial strains were grown at optimal (22°C) and low (4°C) temperatures, and OD_600_ values of the cultures were recorded at regular intervals and plotted against time. All strains displayed optimal growth pattern at 22°C (Fig. 5a). As expected, the cold-sensitive *∆rnr* mutant did not display any measurable growth at 4°C; however, the cold sensitive phenotype of *P. syringae ∆rnr* mutant was rescued by *E. coli*-specific RNase R^Ec^ in the complemented mutant (*∆rnr*/pGL*rnr*^Ec^) (Fig. 5b). The results confirmed that the *E. coli* specific RNase R^Ec^ is active in *P. syringae*, and the enzyme is capable of complementing the functions of *P. syringae* RNase R^Ps^ at low temperature. The activity of the RNase R^Ec^ was comparable to the endogenous RNase R^Ps^, as the growth profiles of the two complemented mutants (*∆rnr*/pGL*rnr*^Ec^ and *∆rnr*/pGL*rnr*^Ps^) were similar.

### Complementation of cold-sensitive phenotype of ∆*rnr *mutant by catalytic domain (RNB^Ec^)

**Fig. 6:**
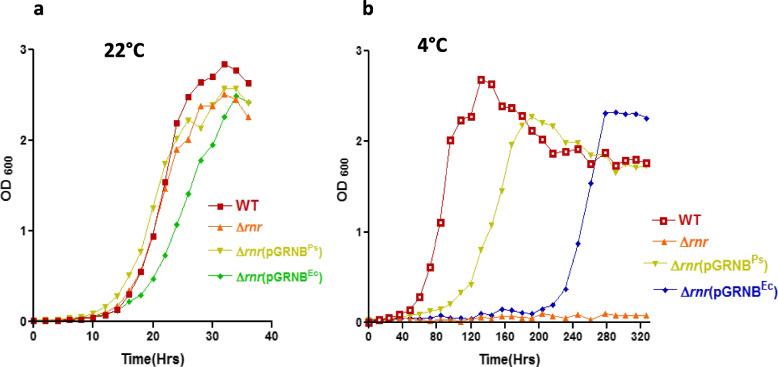
Complementation of ∆*rnr* strain by catalytic domain (RNB) of *E. coli* RNase R. (**a**) Growth analysis of *P. syringae* wild type, ∆*rnr*, ∆*rnr*pGLRNB^Ps^, and ∆*rnr*pGLRNB^Ec ^strains at 22°C and (**b**) at 4°C established that complementation of cold-sensitive ∆*rnr* strain by RNB^Ec^ alleviates the cold-sensitive phenotype of mutant strain but with a long Lag phase even longer than with RNB^Ps^. For measurement of growth in cell cultures at 22°C or 4°C, samples were collected from each culture at regular intervals, and their OD at 600 nm was recorded and plotted against time. Each growth curve was repeated at least three times

To assess the biological activity of the catalytic domain (RNB^Ec^) of *E. coli*-specific RNase R^Ec^ by complementation analysis, we expressed the RNB^Ec^ domain in *P. syringae ∆rnr* mutant from a broad host range plasmid pGL10. For biological activity, growth profiles of *∆rnr* strain (*∆rnr*/pGLRNB^Ec^) expressing the RNB^Ec^ of *E. coli* RNase R were compared with *∆rnr* expressing *P. syringae* RNB (*∆rnr*/pGLRNB^Ps^) and the wild-type. The above strains were grown at 22°C and 4°C, and optical densities of the cultures were measured at OD_600_ at regular intervals and plotted against time. All strains displayed normal growth at 22°C (Fig. 6a). At 4°C, *∆rnr* mutant displayed a cold-sensitive phenotype, whereas the complemented mutants *∆rnr*/pGLRNB^Ps^ and *∆rnr*/pGLRNB^Ec^ displayed measurable growth to stationary phase but with a long lag time (Fig. 6b). The lag time was longer in *∆rnr*/pGLRNB^EC^ compared to *∆rnr*/pGLRNB^PS^ strain. The results confirmed that catalytic domains of both the mesophilic and psychrophilic exoribonuclease R (RNase R) are physiologically active and sufficient for rescuing the cold-sensitive phenotype of *P. syringae ∆rnr* mutant. At low temperatures (4°C), the longer growth lag exhibited by *∆rnr* mutant complemented by RNB^Ec^, compared to *∆rnr* mutant expressing the RNB^Ps^ might be related to the physiological efficiency of RNB^Ps^ over the heterologous *E. coli* specific RNB^Ec^.

## Discussion

The major focus of the current study was to examine the activity of *E. coli* RNase R in the cold-adapted Antarctic *P. syringae*, as RNase R plays an important role in the growth and viability of the *P. syringae*, especially at low temperatures (4°C). RNase R functions inside the cells either as an integral component of a multi-enzyme complex or as a freely soluble enzyme. This is important, as bacteria possess a huge RNA degrading multi-enzyme complex (degradosome) for efficient processing and degradation of different RNA substrates with variable complexity. The RNA degradosome is generally composed of Endo-ribonuclease E (RNase E), that acts as a scaffold for the assembly of other components like exoribonucleases (e.g., PNPase and RNase R), RNA helicases (e.g., RhlB, RhlE, and Rho factors), regulatory proteins (e.g., metabolic enzymes enolase and aconitase) and many transient proteins (e.g., DnaK, GroEL, GroES, Hfq, poly(A) polymerase [42] and polyphosphate kinase, etc.) that functionally interact with each other for the efficient processing and degradation of the substrates. The degradosome is a highly dynamic structure which undergoes changes in composition under different growth conditions within the cells of a species and in different bacterial species with diverged adaptability to different environmental stresses. This has been achieved by the binding activity of highly variable and intrinsically disordered regions of the C-terminal domain of RNase E. In *E. coli* degradosome, C-terminal region of RNase E acts not only as a scaffold for the assembly of exoribonuclease PNPase, RNA helicase RhlB, and the glycolytic enzyme enolase [43, 44] but also helps in localizing the complex to plasma membrane using a membrane targeting sequence (MTS) motif on this domain [45]. Since Antarctic *P. syringae* is adapted to grow at low temperatures, the degradosomal components include RNase E that provides a scaffold for the assembly of the ds-RNA degrading exoribonuclease RNase R, and RNA helicase [46]. Disruption of *rnr* gene leads to defects in the processing of rRNA (16S rRNA and 5S rRNA) and consequent cold-sensitive phenotype accompanied by cell death [30]. On the other hand, the disruption of *rnr* gene in *E. coli* has no deleterious effect on the processing of RNA or growth, although double mutant of *rnr* and *pnp* is not viable [13]. This study has convincingly proved that in *P. syringae,* the important functions of exoribonuclease R in rRNA processing and maintenance of cellular physiology are independent of the degradosomal complex.

Maintaining the rate of enzyme-catalyzed reactions at an acceptable limit to sustain growth by synthesizing cold-active and thermo-labile enzymes in cold environments is the most important adaptation of psychrophiles [46–51]. Lack of strong selective pressure for structural stability in cold environments probably helped in the evolution of cold-active enzymes with increased destabilization and flexibility of active site or whole protein [52]. Reactions catalyzed by cold-active enzymes progress with decreased ∆G and ∆H reflecting that these enzymes are more efficient with high specific activity and are less temperature dependent [53–56]. The active site of these enzymes is less stable and heat-labile [57, 58] whereas these enzymes unfold at relatively lower temperatures than their mesophilic homologs [59]. It has been observed that the majority of cold-adapted enzymes have a half-life of less than 12 min at 50 °C [49, 60, 61]. Cold-adapted enzymes are prone to increased error in folding [62] and cold denaturation, most probably caused by the hydration of polar and non-polar groups [59, 63, 64]. Psychrophilic enzymes are also accompanied by structural changes outside the active site that modulate the activity of critical residues at freezing temperatures and enhance the flexibility of loops around the active site. Cold active enzymes have more accessible and large-sized active sites [65, 66], better channeling of the substrate to the active site, enhanced electrostatic potential, and better release of products [53, 65–69].

Recent studies have provided valuable insights into the structure–function relationship of various enzymes including the ribonucleases. These studies have provided the role of structural flexibility, side chain flexibility, and the role of hydrophobic amino acids in the functioning of the protein. Our results have provided an important input that, despite having variable structural adaptability and substrate specificity, conservation of function allowed the mesophilic enzyme to perform its function in an evolutionary distinct cold-adapted species of bacteria. The role of different polar/non-polar amino acids, amino acid side chains, and structural flexibility may be more related to protein thermostability, thermolability, substrate specificity, and catalysis. Our study has comprehensively proved that exoribonuclease R has a flexible structure that allows it to interact even with less specific substrates and perform its function at a physiologically acceptable rate.

In the light of the differences between two types of degradosomal assembly exemplified by the *E. coli* and *P. syringae*, variations in structure–function relationship among cold-adapted and mesophilic enzymes, differences in biochemical properties and divergent in vivo functions of two exoribonucleases, two questions were raised: (i) will mesophilic *E. coli* RNase R (RNase R^Ec^) be able to complement the cold-sensitive growth defect of *P. syringae* Δ*rnr* mutant? (ii) Despite bearing differences in in vitro activities, will the catalytic domain (RNB^Ec^) of *E. coli* RNase R be able to carry out similar in vivo functions like the catalytic domain (RNB^Ps^) of *P. syringae* RNase R?

Our results illustrate that *E. coli* RNase R (RNase R^Ec^) is capable of rescuing the cold-sensitive growth defect of *P. syringae* Δ*rnr* mutant. The findings of the current study also illustrate that the catalytic domains of two exoribonucleases from *E. coli* and *P. syringae* (RNB^Ec^ and RNB^Ps^) are also capable of complementing the growth defects of *P. syringae* Δ*rnr* mutant at low temperature (4°C). However, *P. syringae* Δ*rnr* mutant expressing the catalytic domain derived from *E. coli* RNase R (RNB^Ec^) displays a longer lag time (~ 240 hours) as compared to the lag time (~ 120 hours) displayed by the *P. syringae* Δ*rnr* mutant complemented by catalytic domain derived from the *P. syringae* RNase R (RNB^Ps^). The difference in the catalytic efficiencies of RNB^Ps^ and RNB^Ec^ at low temperatures is probably related to the substrate specificity, substrate binding, and structural flexibility associated with the two catalytic domains.

Altogether, the results presented here suggest that *E. coli* RNase R could alleviate the cold-sensitive phenotype of *P. syringae* ∆*rnr* mutant at 4°C. The activities of the conserved catalytic domains (RNB^Ps^ and RNB^Ec^) are largely intact in the two diverged bacteria (*P. syringae* and *E. coli*) adapted to grow in different temperature ranges. These results also provide valuable insights into the flexibility of protein structure, structure–function relationship, and conservation of function among the exoribonucleases. Since cold-sensitive ∆*rnr* mutant accumulates unprocessed 5S and 16S rRNA at low temperatures, the rescue of cold sensitivity in ∆*rnr* mutant by RNase R^Ec^ is indicative of an essential role being played by *E. coli* RNase R in 16S and 5S rRNA processing [30, 31].

### Supplementary Information

**Additional file 1:** **Table S1.** Functional activity of *E. coli* RNase R in the Antarctic *Pseudomonas syringae *Lz4W.

## Data Availability

All data generated or analyzed during this study are included in this published article.

## Abbreviations

RNase R: Exoribonuclease R
RNase R^Ec^: *E. coli*-Specific RNase R
RNase R^Ps^: *P. syringae-*Specific RNase R
RNB^Ps^: Catalytic domain of RNase R [*P. syringae*-Specific]
RNB^Ec^: Catalytic domain of RNase R [ *E. coli* specific]
tmRNA: Transfer-messenger RNA

## References

[1] Chu L-Y (2017). Structural insights into RNA unwinding and degradation by RNase R. Nucleic Acids Res.

[2] Hossain ST, Malhotra A, Deutscher MP (2016). How RNase R Degrades structured RNA: Role Of The Helicase Activity And The S1 Domain. J Biol Chem.

[3] Cheng ZF, Deutscher MP (2005). An important role for RNase R in mRNA decay. Mol Cell.

[4] Suzuki H (2006). Characterization of RNase R-digested cellular RNA source that consists of lariat and circular RNAs from pre-mRNA splicing. Nucleic Acids Res.

[5] Matos RG (2011). Swapping the domains of exoribonucleases RNase II and RNase R: conferring upon RNase II the ability to degrade ds RNA. Proteins.

[6] Cairrao F, Arraiano CM (2006). The role of endoribonucleases in the regulation of RNase R. Biochem Biophys Res Commun.

[7] Cheng ZF, Deutscher MP (2002) Purification and characterization of the Escherichia coli exoribonuclease RNase R. Comparison with RNase II. J Biol Chem 277(24):21624–9 10.1074/jbc.M202942200 11948193

[8] Vincent HA, Deutscher MP (2006). Substrate recognition and catalysis by the exoribonuclease RNase R*. J Biol Chem.

[9] Matos RG, Barbas A, Arraiano CM (2009). RNase R mutants elucidate the catalysis of structured RNA: RNA-binding domains select the RNAs targeted for degradation. Biochem J.

[10] Vincent HA, Deutscher MP (2009) The roles of individual domains of RNase R in substrate binding and exoribonuclease activity. The nuclease domain is sufficient for digestion of structured RNA. J Biol Chem 284(1):486–494 10.1074/jbc.M806468200 PMC2610503 19004832

[11] Domingues S (2015). The role of RNase R in trans-translation and ribosomal quality control. Biochimie.

[12] Awano N (2010). Escherichia coli RNase R Has Dual Activities, Helicase and RNase. J Bacteriol.

[13] Cheng ZF, Deutscher MP (2003). Quality control of ribosomal RNA mediated by polynucleotide phosphorylase and RNase R. Proc Natl Acad Sci U S A.

[14] Sulthana S, Deutscher MP (2013). Multiple exoribonucleases catalyze maturation of the 3' terminus of 16S ribosomal RNA (rRNA). J Biol Chem.

[15] Tejada-Arranz A (2021). RNase R is associated in a functional complex with the RhpA DEAD-box RNA helicase in Helicobacter pylori. Nucleic Acids Res.

[16] Andrade JM, Cairrao F, Arraiano CM (2006). RNase R affects gene expression in stationary phase: regulation of ompA. Mol Microbiol.

[17] Venkataraman K, Zafar H, Karzai AW (2014). Distinct tmRNA sequence elements facilitate RNase R engagement on rescued ribosomes for selective nonstop mRNA decay. Nucleic Acids Res.

[18] Cairrao F (2003). Cold shock induction of RNase R and its role in the maturation of the quality control mediator SsrA/tmRNA. Mol Microbiol.

[19] Liang W, Deutscher MP (2010). A novel mechanism for ribonuclease regulation: transfer-messenger RNA (tmRNA) and its associated protein SmpB regulate the stability of RNase R. J Biol Chem.

[20] Moreira RN (2012). Synergies between RNA degradation and trans-translation in Streptococcus pneumoniae: cross regulation and co-transcription of RNase R and SmpB. BMC Microbiol.

[21] Erova TE (2008). Cold shock exoribonuclease R (VacB) is involved in Aeromonas hydrophila pathogenesis. J Bacteriol.

[22] Tobe T (1992). vacB, a novel chromosomal gene required for expression of virulence genes on the large plasmid of Shigella flexneri. J Bacteriol.

[23] Bárria C (2022). RNase R, a new virulence determinant of Streptococcus pneumoniae. Microorganisms.

[24] Charpentier X (2008). Loss of RNase R induces competence development in Legionella pneumophila. J Bacteriol.

[25] Abula A (2021). Molecular mechanism of RNase R substrate sensitivity for RNA ribose methylation. Nucleic Acids Res.

[26] Lalonde MS (2007). Exoribonuclease R in Mycoplasma genitalium can carry out both RNA processing and degradative functions and is sensitive to RNA ribose methylation. RNA.

[27] Hutchison CA (1999). Global transposon mutagenesis and a minimal Mycoplasma genome. Science.

[28] Purusharth RI (2005). Exoribonuclease R interacts with endoribonuclease E and an RNA helicase in the psychrotrophic bacterium Pseudomonas syringae Lz4W. J Biol Chem.

[29] Shivaji S, et al (1989) Isolation and identification of Pseudomonas spp. from Schirmacher Oasis, Antarctica. Appl Environ Microbiol 55(3):767–70 10.1128/aem.55.3.767-770.1989 PMC184196 2930174

[30] Purusharth RI, Madhuri B, Ray MK (2007). Exoribonuclease R in Pseudomonas syringae is essential for growth at low temperature and plays a novel role in the 3' end processing of 16 and 5 S ribosomal RNA. J Biol Chem.

[31] Sulthana S, Basturea GN, Deutscher MP (2016). Elucidation of pathways of ribosomal RNA degradation: an essential role for RNase E. RNA.

[32] Janiyani KL, Ray MK (2002). Cloning, sequencing, and expression of the cold-inducible hutU gene from the antarctic psychrotrophic bacterium Pseudomonas syringae. Appl Environ Microbiol.

[33] Regha K, Satapathy AK, Ray MK (2005). RecD plays an essential function during growth at low temperature in the antarctic bacterium Pseudomonas syringae Lz4W. Genetics.

[34] Malke H, Sambrock J, Fritsch EF, Maniatis T (1989) Molecular Cloning, A Laboratory Manual (Second Edition), Volumes 1, 2 and 3. 1625 S., zahlreiche Abb. und Tab. Cold Spring Harbor: Cold Spring Harbor Laboratory Press. $ 115.00. ISBN: 0–87969–309–6. 1990. 30(8): p. 623–623

[35] Strand TA (2014). A new and improved host-independent plasmid system for RK2-based conjugal transfer. PLoS ONE.

[36] Liu J (2018). An improved method of preparing high efficiency transformation Escherichia coli with both plasmids and larger DNA fragments. Indian J Microbiol.

[37] Froger A, Hall JE (2007) Transformation of plasmid DNA into E. coli using the heat shock method. J Vis Exp 6:253 10.3791/253 PMC2557105 18997900

[38] Bidle KA, Bartlett DH (1999). RecD function is required for high-pressure growth of a deep-sea bacterium. J Bacteriol.

[39] Sulthana S (2011). rnr gene from the antarctic bacterium Pseudomonas syringae Lz4W, encoding a psychrophilic RNase R. Appl Environ Microbiol.

[40] Studier FW, Moffatt BA (1986). Use of bacteriophage T7 RNA polymerase to direct selective high-level expression of cloned genes. J Mol Biol.

[41] Sinha AK (2013). Replication arrest is a major threat to growth at low temperature in Antarctic Pseudomonas syringae Lz4W. Mol Microbiol.

[42] Braman J, Papworth C, Greener A (1996). Site-directed mutagenesis using double-stranded plasmid DNA templates. Methods Mol Biol.

[43] Carpousis AJ (2002). The Escherichia coli RNA degradosome: structure, function and relationship in other ribonucleolytic multienzyme complexes. Biochem Soc Trans.

[44] Worrall JA (2008). Reconstitution and analysis of the multienzyme Escherichia coli RNA degradosome. J Mol Biol.

[45] Khemici V (2008). The RNase E of Escherichia coli is a membrane-binding protein. Mol Microbiol.

[46] Ponnada PSk, et al (2011) Cold active enzymes from the marine psychrophiles: biotechnological perspective. Adv Biotech 10:16–20

[47] Åqvist J, Isaksen GV, Brandsdal BO (2017). Computation of enzyme cold adaptation. Nat Rev Chem.

[48] Gerday C (2014) Fundamentals of cold-active enzymes, in cold-adapted yeasts: biodiversity, adaptation strategies and biotechnological significance. Buzzini P, Margesin R, Editors. Springer Berlin Heidelberg, Berlin, 325–350

[49] Siddiqui KS, Cavicchioli R (2006). Cold-adapted enzymes. Annu Rev Biochem.

[50] Lonhienne T, Gerday C, Feller G (2000). Psychrophilic enzymes: revisiting the thermodynamic parameters of activation may explain local flexibility. Biochim Biophys Acta.

[51] Rishi N, Goel R (2022). Enzymatic Behaviour of Cold Adapted Microbes. Survival Strategies in Cold-adapted Microorganisms.

[52] Gerday C (2000). Cold-adapted enzymes: from fundamentals to biotechnology. Trends Biotechnol.

[53] Collins T, et al (2008) Fundamentals of Cold-Adapted Enzymes, in Psychrophiles: from Biodiversity to Biotechnology, R. Margesin, et al., Editors. Springer Berlin Heidelberg, Berlin, p. 211–227

[54] Deniz AA (2018). Enzymes can adapt to cold by wiggling regions far from their active site. Nature.

[55] Peterson ME (2007). The dependence of enzyme activity on temperature: determination and validation of parameters. Biochem J.

[56] Daniel RM (2008). The effect of temperature on enzyme activity: new insights and their implications. Extremophiles.

[57] Marx JC (2007). Cold-adapted enzymes from marine Antarctic microorganisms. Mar Biotechnol (NY).

[58] Sočan J, Purg M, Åqvist J (2020). Computer simulations explain the anomalous temperature optimum in a cold-adapted enzyme. Nat Commun.

[59] D'Amico S (2003). Activity-stability relationships in extremophilic enzymes. J Biol Chem.

[60] Chen Y, Tian Q, Wang H, Ma R, Han R, Wang Y et al (2022) A manganese-based metal-organic framework as a cold-adapted nanozyme. Adv Mater e2206421. 10.1002/adma.202206421 10.1002/adma.202206421 36329676

[61] Georlette D (2004). Some like it cold: biocatalysis at low temperatures. FEMS Microbiol Rev.

[62] D'Amico S, Gerday C, Feller G (2001). Structural determinants of cold adaptation and stability in a large protein. J Biol Chem.

[63] Makhatadze GI, Privalov PL (1995). Energetics of protein structure. Adv Protein Chem.

[64] Aurilia V, et al (2009) Structure and dynamics of cold-adapted enzymes as investigated by FT-IR spectroscopy and MD. The Case of an Esterase from Pseudoalteromonas haloplanktis. J Phys Chem B 113(22):7753–7761 10.1021/jp901921r 19435327

[65] Aghajari N (2003). Crystal structures of a psychrophilic metalloprotease reveal new insights into catalysis by cold-adapted proteases. Proteins.

[66] Russell RJ (1998). Structural adaptations of the cold-active citrate synthase from an Antarctic bacterium. Structure.

[67] Khan S, Farooq U, Kurnikova M (2016). Exploring protein stability by comparative molecular dynamics simulations of homologous hyperthermophilic, mesophilic, and psychrophilic proteins. J Chem Inf Model.

[68] Kim SY, et al (1999) Structural basis for cold adaptation. Sequence, biochemical properties, and crystal structure of malate dehydrogenase from a psychrophile Aquaspirillium arcticum. J Biol Chem 274(17):11761–7 10.1074/jbc.274.17.11761 10206992

[69] Smalås AO (2000). Cold adapted enzymes. Biotechnol Annu Rev.

