# Supplementary material for: Functional activity of E. coli RNase R in the Antarctic Pseudomonas syringae Lz4W
Source: J Genet Eng Biotechnol. 2023 Oct 16;21:101. doi: 10.1186/s43141-023-00553-2 (PMC10579198; doi:10.1186/s43141-023-00553-2)
Supplement: Supplementary file 1 — Additional file 1: Table S1. Functional activity of E. coli RNase R in the Antarctic Pseudomonas syringae Lz4W. [file 43141_2023_553_MOESM1_ESM.docx]

**Supplementary information to**

**Functional activity of *E. coli* RNase R in the Antarctic *Pseudomonas syringae* Lz4W**

**Table S1.**

| **Primer** | **Sequence (5′**-**3′)** | **Description** |
| --- | --- | --- |
| ERNRFP ERNRRP | ACCAGGCATATGTCACAAGATCCTTTCCAGGAACG AACTCACGTCGACTCACTCTGCCACTTTTTTCTTCG | *E. coli rnr* FP and RP |
| ECRNBFP ECRNBRP | GGCACCCATATGGTCGAAGTGCTGGGCGAC TAAAGGGATCCTTACTGGTCGAGCATGAAGTCACAC | *E. coli* RNB FP and RP |
